# Effect of a high crude protein content diet during energy restriction and re-alimentation on animal performance, skeletal growth and metabolism of bone tissue in two genotypes of cattle

**DOI:** 10.1371/journal.pone.0247718

**Published:** 2021-02-25

**Authors:** Tiago A. C. C. Silva, Simon P. Quigley, Lisa J. Kidd, Stephen T. Anderson, Stuart R. McLennan, Dennis P. Poppi

**Affiliations:** 1 School of Environmental and Rural Science, University of New England, Armidale, Australia; 2 School of Agriculture and Food Sciences, The University of Queensland, Gatton, Australia; 3 School of Veterinary Science, The University of Queensland, Gatton, Australia; 4 School of Biomedical Sciences, The University of Queensland, St Lucia, Australia; 5 Centre for Animal Science, Queensland Alliance for Agriculture and Food Innovation, The University of Queensland, Dutton Park, Australia; University of Life Sciences in Lublin, POLAND

## Abstract

The objective of this study was to investigate the effect of diet crude protein (CP) content and metabolisable energy (ME) intake on skeletal growth and associated parameters of growing steers prior to and during compensatory growth in weight and catch-up growth in skeletal elongation. The experiment was a factorial design with two cattle genotypes [Brahman crossbred (BX, 178 ± 6 kg) and Holstein-Friesian (HF, 230 ± 34 kg)] and three nutritional treatments; high CP content and high ME intake (HCP-HME), high CP content and low ME intake (HCP-LME) and low CP content and low ME intake (LCP-LME) with the ME intake of HCP-LME matched to that of LCP-LME. Nutritional treatments were imposed over a 103 d period (Phase 1), and after this, all steers were offered *ad libitum* access to the HCP-HME nutritional treatment for 100 d (Phase 2). Steers fed the high CP content treatment with a low ME intake, showed higher hip height gain (*P* = 0.04), larger terminal hypertrophic chondrocytes (*P* = 0.02) and a higher concentration of total triiodothyronine in plasma (*P* = 0.01) than steers with the same ME intake of the low CP content treatment. In addition, the low CP treatment resulted in significant decreases in bone volume (*P* = 0.03), bone surface area (*P* = 0.03) and the concentration of bone-specific alkaline phosphatase in plasma (*P* < 0.001) compared to steers fed the HCP-HME treatment. A significant interaction between genotype and nutritional treatment existed for the concentration of thyroxine (T4) in plasma where HF steers fed LCP-LME had a lower T4 concentration in plasma (*P* = 0.05) than BX steers. All steers with a restricted ME intake during Phase 1 demonstrated compensatory growth during Phase 2. However, HF steers fed the LCP treatment during Phase 1 showed a tendency (*P* = 0.07) for a greater LWG during Phase 2 without any increase in dry matter intake. Results observed at the growth plate and hip height growth suggest that catch-up growth in cattle may also be explained by the growth plate senescence hypothesis. Contrary to our initial hypothesis, the results demonstrate that greater CP intake during ME restriction does not increase compensatory gain in cattle during re-alimentation.

## Introduction

Skeletal growth through passive stretching accounts for increases in muscle volume, sarcomere numbers and cross-sectional area of myofibers [1–3]. Muscles and organs will always tend to maintain allometric relationship with the skeleton [4]. Compensatory growth is the term usually adopted in animal production, and elsewhere in this paper, to describe the process whereby animals demonstrate a faster liveweight (LW) gain (LWG) when returning to a “normal” weight-for-height relationship [5]. The term catch-up growth is commonly used in the medical literature [6, 7] but also in some areas of animal physiology [8, 9], and will be adopted here to describe a faster skeletal growth rate of previously restricted animals when returning to a normal height-for-age. Nevertheless, these two terms (i.e. compensatory and catch up growth) are sometimes adopted as synonymous [10–12] and in other instances treated as separated phenomenon’s based on the growth curves obtained after restoration of non-restrictive conditions [13]. In the present study we will define compensatory growth as the process whereby the body compensates for deviations in the relationship between soft tissue (i.e. muscles and organs) and skeleton size. Catch-up growth may be defined as the process where restricted animals demonstrate increased skeletal growth when compared to an aged matched unrestricted cohort. During catch-up growth greater skeletal growth is explained by the growth plate delayed senescence hypothesis [14–17].

The somatotropic axis is the major endocrine regulator of endochondral ossification and bone turnover [18]. Increased intake of crude protein (CP) stimulates insulin-like growth factor 1 (IGF-1) in cattle but the IGF-1 response to CP intake is decreased when metabolizable energy (ME) intake is low [19]. However, it is unknown if greater CP intake will stimulate IGF-1 production during a severe ME restriction in cattle. Studies with rodents have shown that endochondral ossification and trabecular bone turnover can be stimulated by increased protein intake even in situations of energy restriction [20–22].

The hypothesis of the current study was that a higher CP intake during energy restriction will increase the concentration of IGF-1 in plasma and stimulate bone elongation. A greater skeletal size with lower LW at the end of a restricted period of nutrition will result in greater LWG during compensatory growth in the subsequent wet season. In addition, the rates of skeletal growth and LWG are expected to differ between different genotypes, however endocrinal changes should respond similarly to nutritional changes independent of genotype. Therefore, the objectives of this experiment were to investigate the effect of a higher CP content diet during energy restriction and re-alimentation on performance and skeletal growth of two genotypes of cattle.

## Materials and methods

The experiment was conducted in accordance with the Australian Code of Practice for the Care and Use of Animals for Scientific Purposes and was approved by The University of Queensland Animal Ethics Committee (SVS/171/12/MLA).

### Experimental design and animal management

Fifteen Brahman crossbred (BX; 5/8 *Bos indicus* and 3/8 *Bos taurus*) steers and fifteen Holstein-Friesian (HF) steers were fed a Rhodes (*Chloris gayana*) grass and Dolichos lablab (*Lablab purpureus*) mixed hay [905 g organic matter (OM), 88 g CP, 644 g ash-free neutral detergent fiber (NDF), 3.9 g P and 7.9 g Ca/kg dry matter (DM)] *ad libitum* in group pens for 23 d and in individual pens for 7 d prior to the commencement of the experiment.

At the commencement of the experiment (d 1) the HF steers (230 ± 34 kg LW) and BX steers (178 ± 6 kg) were ranked and blocked on LW from heaviest to lightest within each genotype and allocated to one of five blocks of adjacent individual pens (n = 6 pens/block). Three steers from the same LW ranking block within each genotype were randomly allocated to individual pens within each block of pens. For each block of pens the steers from each genotype were then randomly allocated one of three nutritional treatments, high CP content and high ME intake (HCP-HME), high CP content and low ME intake (HCP-LME) and low CP content and low ME intake (LCP-LME). Each individual steer was considered as the experimental unit.

The experiment consisted of two phases. During Phase 1 (103 d duration) the steers were offered one of the three nutritional treatments: HCP-HME, HCP-LME and LCP-LME. The HCP-HME and HCP-LME treatments consisted of lucerne [*Medicago sativa*; 896 g OM, 9.7 MJ ME, 199 g CP, 356 g NDF, 271 g ash-free acid detergent fibre (ADF), 3.2 g P and 12.2 g Ca/kg DM] chaffed hay (chopped to approximately 5 to 10 mm in length) and the LCP-LME treatment consisted of Mitchell grass (*Astrebla* spp.; 901 g OM, 5.1 MJ ME, 38 g CP, 631 g NDF, 248 g ADF, 1.8 g P and 4.7 g Ca/kg DM) hay [chopped to approximately 50 mm in length (Jaylor 4350 Feed Grinder, McIntosh and Son; Dalby, QLD, Australia)]. The HCP-HME and LCP-LME treatments were offered *ad libitum* and the daily quantity offered was calculated based on the previous day’s intake plus 10% and 20% respectively on an as-fed basis. The feed allowance for steers allocated to the HCP-LME treatment was calculated from the mean ME intake of all steers within the corresponding genotype allocated to the LCP-LME treatment during the previous week in such a manner that steers allocated to the HCP-LME and LCP-LME treatments had the same ME intake but different CP intake. Metabolizable energy content of each nutritional treatment was estimated by the equation M/D = 0.172DMD– 1.707, where M/D represents the metabolizable energy content per kilo of DM and DMD stands for dry matter digestibility as described in Freer et al. [23]. Steers allocated to the LCP-LME treatment were offered 50 g of cottonseed meal (CSM; 924 g OM, 42.8 g CP, 237 g NDF, 14.6 g P and 2.5 g Ca/kg DM) per kilo of hay (as-fed basis) from d 42 until the end of Phase 1 and steers fed HCP-LME treatment were offered 84 mg mono-sodium phosphate (MSP; 240 g P/kg DM) per kilo of LW daily. During Phase 2 (100 d duration) all steers were offered the same lucerne chaff used in Phase 1 *ad libitum* (i.e. HCP-HME) and the ME content estimated in Phase 1 was used in Phase 2 calculations.

Feed residues were collected and weighed at 0730 h and steers were offered feed at approximately 0800 h each day. Sub-samples of feed offered were collected daily at feeding and bulked over 7 d. Feed residues were weighed daily and bulked over 7 d for each steer. Duplicate sub-samples of feed offered and feed residues for each steer were collected at the end of each 7 d period.

### Liveweight and hip height measurement

Liveweight was measured prior to feeding on the same day each week throughout the experiment. Hip height (HH) was measured at the highest point of the sacrum between the tuber coxae bones using a measuring stick on d -10, 7, 35, 57, 70, 85, 103, 112, 125, 140, 154, 168, 182, 196 and 203 of the experiment. Liveweight gain (LWG) and HH gain (HHG) were calculated by regressing each measurement over time within each phase of the experiment.

### Microbial crude protein production, digestibility and rumen fluid collection

Two cohorts of steers (n = 15/cohort) were moved into individual metabolism crates on d 19 and 33 of Phase 1 of the experiment in the same sequence as their individual pens. Each cohort remained in the metabolism crates for 9 consecutive days, which included a 2 days adaptation period and a 7 days collection period. Total faecal and urine output and feed residues were collected and weighed daily. Daily urine output of each steer was acidified to pH 3 with 5% sulphuric acid and a 5% sub-sample was collected, stored at 4°C and bulked over the collection period. At the end of the collection period the bulked urine was mixed well and aliquots were stored at -20°C for subsequent analysis. A 10% sub-sample of faecal output was collected daily, stored at 4°C and bulked over the collection period. At the end of the collection period the bulked faeces were mixed well and triplicate sub-samples were collected for subsequent analysis. Triplicate sub-samples of feed offered and residues were also collected for subsequent analysis. Upon the completion of each faecal and urine collection period the steers were returned to their individual pens and rumen fluid was collected prior to feeding per *os* using a stomach tube attached to a hand pump.

### Blood samples

Blood samples were collected from the jugular vein on d -10, 103 (end of Phase 1), and 203 (near the end of Phase 2) into lithium heparin coated vacutainers (Becton Dickinson; Franklin Lakes, NJ, USA). Vacutainers were centrifuged at 1700 *g* for 10 min at 4°C and plasma was collected and stored at -20°C until analysis.

### Tuber coxae bone biopsy

The biopsy site was clipped and scrubbed with chlorhexidine surgical scrub (Perrigo; Balcatta, WA, Australia) and wiped clean with Chlor-hex C (Jurox; Rutherford, NSW, Australia) in methylated spirits (Recochem; Lytton, QLD, Australia). The skin and deeper tissue over the tuber coxae were infiltrated with 35 to 40 mL of lignocaine hydrochloride (20 mg lignocaine hydrochloride/mL; Troy laboratories; Glendenning, NSW, Australia) and left for 5 min for effect. An incision approximately 80 mm in length was made and skin and any overlying muscle were retracted. A single biopsy 10 to 20 mm deep was obtained from the most central part of the tuber coxae of the ilium. A 10 to 20 mm bone hand-trephine (Sontec Instruments Inc.; Centennial, CO, USA) was used to start the biopsy and then a 16 mm metal hole-saw was used to obtain a deeper sample. An elevator was used to separate the sample from the parent bone. Overlying muscle was sutured with absorbable sutures (2/0 PDS) and the external incision closed with skin staples, the surgical site was then cleaned and sprayed with Chloromide antiseptic spray (Troy laboratories; Glendenning, NSW, Australia). The bone cores were divided lengthwise using a scalpel blade and sub-samples were fixed with either 10% neutral buffered formalin (NBF) or 4% paraformaldehyde (PFA) and placed on ice prior to transfer to the laboratory. The samples remained in fixative for 24 h at 4°C and were then transferred to 70% ethanol and stored at 4°C for approximately 4 months until they were sectioned.

Bone biopsies were collected from the tuber coxae on the left, right and left side of each steer on d -10, 104 and 207 of the experiment respectively. On d 104 the biopsies were collected before the change in diet from Phase 1 to 2. Collection of intake and body dimension data concluded on d 203 but steers were maintained on the same Phase 2 diet until d 207.

### Laboratory analysis

Sub-samples of feed offered and feed residues were dried in duplicate to a constant weight at 65°C for DM determination. Samples were then ground through a 1 mm screen (Retsch ZM 200; Haan, Germany), dried for 24 h at 105°C to determine residual DM content and then combusted in an electric muffle furnace (Modutemp Pty. Ltd.; Perth, WA, Australia) for 8 h at 550°C to determine organic matter (OM) content. The N content of feeds was measured by the Kjeldahl method using an auto-digestor (Tecator 2520, FOSS; Hillerød, Denmark) and a N analyser (Kjeltec 8400, FOSS; Hillerød, Denmark) following the manufacturers guidelines. Crude protein content was calculated using the conversion factor 6.25 x N. The content of ash-free NDF and ADF in feeds was measured following the procedure described by Van Soest et al. [24] using a fiber analyzer (A200, Ankom; Macedon, NY, USA). The mineral content of feeds was determined on an ICP-OES spectrometer (Optima 7300 DV, PerkinElmer; Waltham, MA, USA) after a nitric-perchloric acid digestion.

The concentration of glucose, nonesterified fatty acids (NEFA), calcium, inorganic phosphorus, urea-N (PUN), total protein, insulin, IGF-1, total triiodothyronine (T3), total thyroxine (T4), leptin, bone-specific alkaline phosphatase (BALP), osteocalcin (OCN), pyridinoline crosslinks (PYD) and total deoxypyridinoline crosslink (tDPD) in plasma were determined using a rage of commercially available colorimetric, radioimmuno and enzyme linked assays (S1 Appendix).

Thawed urine sub-samples were filtered through a 0.2 μm membrane (Phenex-RC, Phenomenex, Torrance, CA, USA) and the concentration of purine derivatives was determined using a high-performance liquid chromatograph method described by Balcells et al. [25]. Microbial crude protein (MCP) production was then estimated using the method of Chen and Gomes [26] using the endogenous purine derivative values for *Bos indicus* and *Bos taurus* cattle described by Bowen et al. [27].

Rumen fluid samples were acidified with 20% sulphuric acid and stored at -20°C. The concentration of NH_3_H was measured by titration with 0.01 *M* HCl using a TIM 840 Titration workstation manager (Radiometer Analytical SAS; Villeubanne Cedex, France) after distillation using a semi-automatic distillation unit (UDK 139, Velp Scientifica; Usmate, MB, Italy). The concentration of individual volatile fatty acids (VFA) in the rumen fluid were analysed by gas liquid chromatography (GC-2010, Shimadzu; Kyoto, Honshu, Japan) fitted with a polar capillary column (ZB-FFAP, Phenomenex; Lane Cove, NSW, Australia).

**Fig 1:**
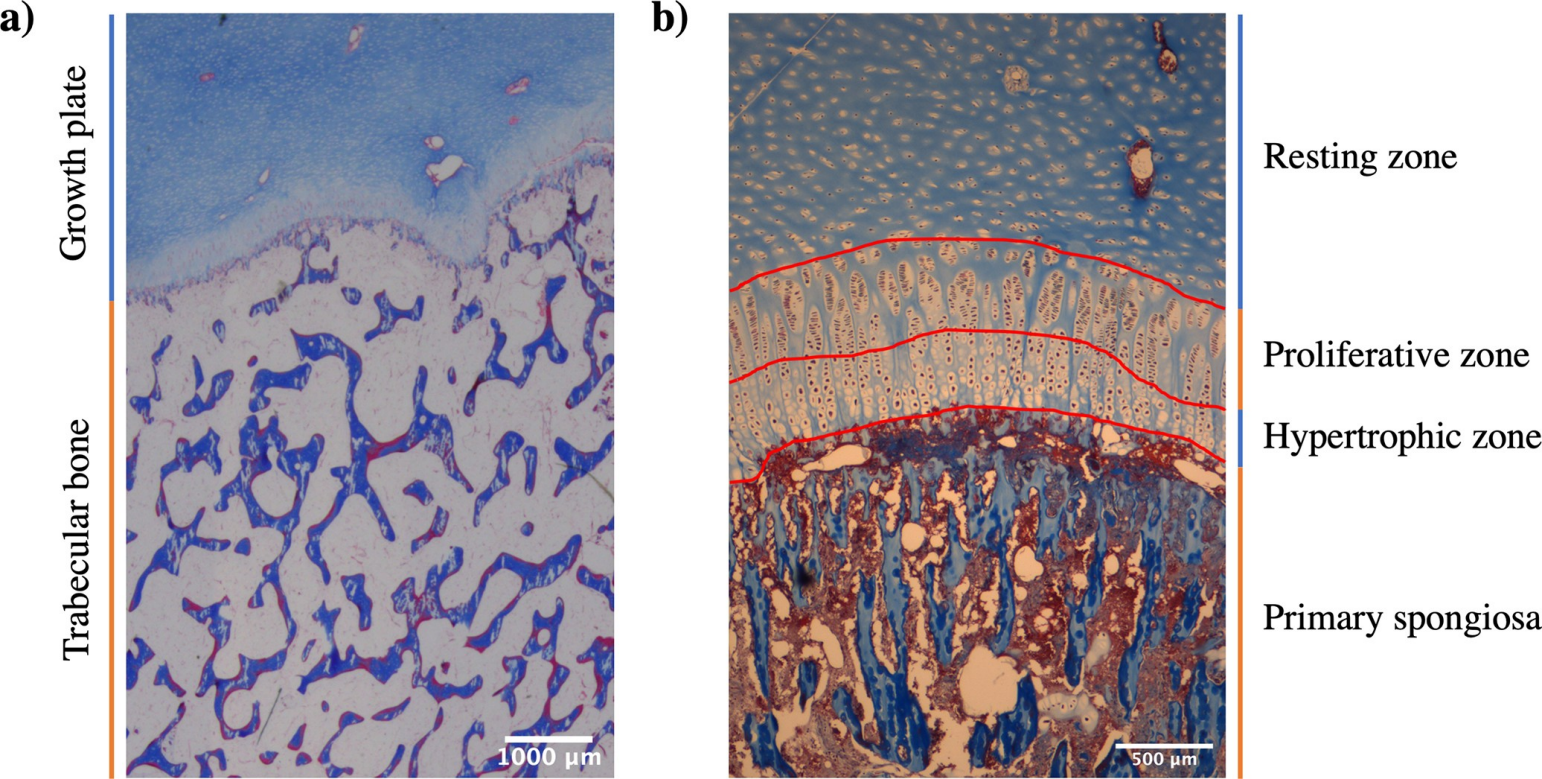
Mason trichrome stained sections of cattle’s growth plate and trabecular of tuber coxae bone and photographed at 1X (a) and 4X (b) objectives. Histomorphometric assessments were conducted at the trabecular bone (a) as well as at the proliferative and hypertrophic zones of the growth plate (b).

Bone samples were decalcified using a 10% EDTA (pH 7.0) solution [28]. The sections were stained with toluidine blue and Masson trichrome and photographed twice with a 1X and 4X objectives using an Olympus BX41 microscope (Olympus America Inc.; Melville, NY, USA) equipped with a digital camera Q-Imaging camera (Qimaging Corporation; Surrey, BC, Canada). The images (Fig 1A and 1B) were then analysed using ImageJ software [29] for determination of proliferative (PZ) and hypertrophic (HZ) zone heights, number of hypertrophic chondrocytes (HC) per column and diameter of terminal hypertrophic chondrocytes (THC). The BoneJ plugin [30] was used to obtain values for bone volume (Bv/Tv), trabecular separation (Tb.Sp), trabecular thickness (Tb.Th) and bone surface (BS) [31]. Changes in bone volume were calculated by the difference between the initial bone volume recorded at the start minus the volume at the end of each phase.

### Statistical analysis

All statistical analyses were conducted using the open-source software R version [32] and the linear mixed models procedure of the package “nlme” [33]. Prior to analysis all data was checked for normality and homoscedasticity and, if necessary, data was transformed according to the Box-Cox procedure [34]. For LWG and HHG the initial LW and HH of each phase were used as covariates. All the other parameters were compared within the time-point at which they were collected. Nutrition and genotype treatments and their interaction were included in the model as fixed effects and steers within block were included as random factors. Tukey *post hoc* test was performed to explore the differences between groups whenever main factors or interactions were significant (*P* < 0.05). Stepwise linear regression analysis was conducted to assess the effect of ME and CP intake on LWG and HHG. The initial models included ME intake, CP intake and its interaction with genotype as explanatory variables and they were removed when not significant (*P* > 0.05). The final models were checked for homoscedasticity, normality and multicollinearity. Metabolisable energy intake and CP intake were highly correlated (r = 0.95) which could violate the collinearity assumption if both variables were included in the final model. However, in none of the models were both variables (i.e. ME and CP intake) shown to be significant simultaneously. Pearson’s correlation coefficient (r) was calculated for the concentration of bone markers in the plasma and the hystomorphometric parameters of the trabecular bone.

## Results

### Rumen parameters and microbial protein production

The manipulation of the diets delivered the expected changes in ME and CP supply. The DM digestibility of the lucerne chaff did not differ (*P* > 0.05) with intake in the current experiment [65.6% and 67.2% at *ad libitum* (HCP-HME) and limited intakes (HCP-LME) respectively] but was significantly greater (*P* < 0.01) than the digestibility of the Mitchell grass hay (40.1%) consumed by steers offered the LCP-LME treatment (Table 1). Digestibility was unaffected by genotype (*P* = 0.11; Table 1). Steers fed the Mitchell grass hay based diet (LCP-LME) had a lower concentration of NH_3_N in the rumen (*P* < 0.001), produced less MCP (*P* < 0.001) and had a lower (*P* < 0.001) efficiency of MCP production [EMCP, g MCP/kg digestible organic matter (DOM)] than steers fed the lucerne chaff based diets (HCP-HME and HCP-LME) regardless of DM and ME intake (Table 1). Holstein-Friesian steers fed the LME-LCP treatment diet had a significantly lower concentration (16.2 *vs* 83.3 mg/L; *P* < 0.005; not shown) of NH_3_N in the rumen than BX steers fed the same nutritional treatment. Microbial protein production but not the EMCP was greater (*P* < 0.001) in steers fed lucerne chaff *ad libitum* (HCP-HME) compared with steers fed a limited amount of lucerne chaff (HCP-LME). Steers with limited DM intake (HCP-LME) had a lower (*P* < 0.001 concentration of VFA in the rumen than steers fed *ad libitum* (HCP-HME and LCP-LME) regardless of the CP content of the diet. The VFA proportions were similar across treatments with only small differences in acetic and propionic acid with the Mitchell grass diet (LME-LCP). Butyric acid concentration was greater in the rumen of steers fed HCP-HME followed by HCP-LME and LCP-LME.

**Table 1:** The Dry Matter Digestibility (DMD), pH, concentration of ammonia (NH3N) and Volatile Fatty Acids (VFA) and the molar percentage of acetic, butyric and propionic acids in the rumen fluid and the microbial protein (MCP) production and the efficiency of MCP production (EMCP) of Holstein-Friesian (HF) and Brahman crossbred (BX) steers fed different nutritional treatments during Phase 1 of the experiment.

Item	Nutrition (N)^1^^,^^2^			Genotype (G)		SEM	*P*-Value		
	HCP-HME	HCP-LME	LCP-LME	HF	BX		N	G	N x G
DMD, %	65.6^b^	67.2^b^	40.1^a^	56.7	58.6	2	<0.001	0.11	0.59
pH	7.20^b^	7.27^b^	6.90^a^	7.14	7.11	0.1	<0.001	0.65	0.16
NH_3_N, mg/L	131.4^b^	130.9^b^	49.7^a^	87	121	11	<0.001	<0.005	0.04
MCP prod., g/kg LW	2.17^c^	0.62^b^	0.32^a^	1.15	0.92	0.1	<0.001	0.77	0.45
EMCP, g MCP/kg DOMI^3^	91.0^b^	72.6^b^	32.2^a^	65.8	64.7	8.5	<0.001	0.88	0.38
Total VFA, mmol/L	47.8^b^	36.5^a^	51.7^b^	45.2	45.4	3.5	<0.001	0.96	0.04
Acetic,%	74.1^a^	74.2^a^	78.1^b^	76.7	74.2	0.7	<0.001	<0.001	0.21
Propionic,%	13.0^a^	12.3^a^	14.4^b^	13.2	13.3	0.4	<0.001	0.76	0.4
Butyric,%	8.4^c^	4.15^b^	2.5^a^	6.5	3.4	0.8	<0.001	<0.001	0.45

^a-c^ Means within a row with different superscripts differ (*P* < 0.05).

^1^ Data are least squares means, with standard error of the mean (SEM).

^2^ Steers were fed high crude protein and high metabolizable energy content (HCP-HME), high CP and low ME content (HCP-LME) and low CP and low ME content (LCP-LME) diets.

^3^ Digestible organic matter intake (DOMI).

### Intake, liveweight gain, hip height gain, feed conversion ratio and liveweight:hip height

**Fig 2:**
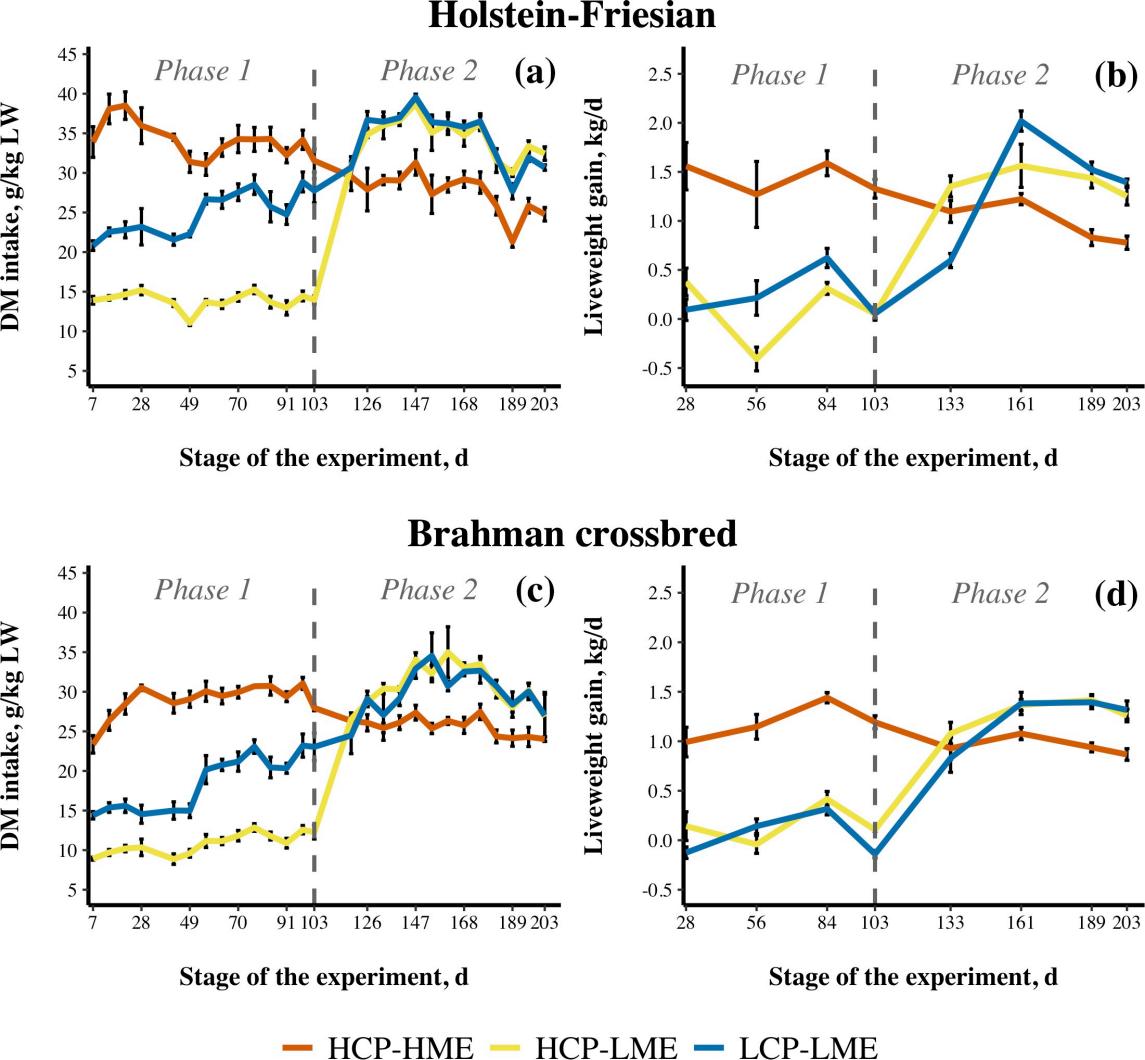
Dry matter (DM) intake and liveweight gain of Holstein-Friesian (a and b) and Brahman crossbred (c and d) steers fed high crude protein and high metabolizable energy content (HCP-HME), high CP and low ME content (HCP-LME) and low CP and low ME content (LCP-LME) diets throughout the experiment. The different nutritional treatments were imposed from day 1 until day 103 (Phase 1), after that all steers were fed HCP-HME *ad libitum* until day 203 (Phase 2). Dashed grey line represents the transition from Phase 1 to Phase 2. Error bars represents standard error of the mean.

During Phase 1 of the experiment, steers fed the HCP-HME treatment had higher (*P* < 0.001) DM, ME and CP intake than steers offered the HCP-LME and LCP-LME treatments (Table 2) while steers offered the LCP-LME treatment had higher DMI (*P* < 0.001), lower CPI (*P* < 0.001) but comparable MEI (*P* = 0.3) to steers offered the HCP-LME treatment, as planned. The HF steers had significantly higher DMI and MEI than BX steers, while CPI and DMD were unaffected by genotype. During Phase 2 of the experiment, steers with restricted MEI during Phase 1 (HCP-LME and LCP-LME) had higher DMI, MEI and CPI (all *P* < 0.001) with HF steers having higher DMI, MEI and CPI than BX steers (all *P* < 0.001). The peak of DM intake was observed approximately 60 d after the beginning of Phase 2 (Fig 2).

**Table 2:** Dry Matter Intake (DMI), Metabolizable Energy (ME) intake (MEI), and Crude Protein (CP) intake (CPI), feed conversion ratio (FCR), liveweight gain (LWG), Hip Height Gain (HHG) and liveweight:hip height (LW:HH) of Holstein-Friesian (HF) and Brahman crossbred (BX) steers fed different nutritional treatments in Phase 1 and re-alimentation (Phase 2).

Item	Nutrition (N)^1^^,^^2^			Genotype (G)		SEM	*P*-Value		
	HCP-HME	HCP-LME	LCP-LME	HF	BX		N	G	N x G
*Phase 1*									
DMI, g/kg LW	31.5^c^	12.1^a^	20.3^b^	23.6	18.9	0.9	<0.001	<0.001	0.06
MEI, MJ/kg LW	0.30^b^	0.12^a^	0.11^a^	0.19	0.16	0.01	<0.001	<0.05	0.46
CPI, g/kg LW	6.13^c^	2.00^b^	0.84^a^	3.15	2.82	0.22	<0.001	0.15	0.06
FCR, kg DMI/kg LWG	6.3^a^	30.2^b^	47.2^b^	29.3	27	11.3	<0.001	0.32	0.1
LWG, kg/d	1.27^b^	0.16^a^	0.14^a^	0.58	0.47	0.06	<0.001	<0.05	0.02
HHG, mm/100 d	104.7^c^	41.8^b^	30.3^a^	63	54.4	6.20	<0.001	<0.05	0.01
LW:HH, kg/cm	2.1^c^	1.5^a^	1.7^b^	1.96	1.65	0.15	<0.001	<0.001	0.41
***Phase 2***									
DMI, g/kg LW	26.6^a^	32.6^b^	32.2^b^	32.1	28.8	0.6	<0.001	<0.001	0.27
MEI, MJ/kg LW	0.25^a^	0.31^b^	0.30^b^	0.3	0.27	0.02	<0.001	<0.001	0.2
CPI, g/kg LW	4.41^a^	5.39^b^	5.34^b^	5.33	4.77	0.17	<0.001	<0.001	0.24
FCR, kg DMI/kg LWG	11.2^a^	6.8^b^	6.1^b^	9.2	6.8	0.49	<0.001	<0.001	0.74
LWG, kg/d	0.93^a^	1.34^b^	1.45^b^	1.3	1.2	0.07	<0.001	0.08	0.04
HHG, mm/100 d	75^a^	89^b^	89^b^	92	77.1	7.00	<0.05	<0.05	0.38
LW:HH, kg/cm	2.9^b^	2.1^a^	2.2^a^	2.6	2.2	0.07	<0.001	<0.001	0.32

^a-c^ Means within a row with different superscripts differ (*P* < 0.05).

^1^ Data are least squares means with standard error of the mean (SEM).

^2^ Steers were fed nutritional treatments with a high crude protein content and high metabolizable energy intake (HCP-HME), high CP content and low ME intake (HCP-LME) and low CP content and low ME intake (LCP-LME) during Phase 1 and were offered the HCP-HME diet *ad libitum* during Phase 2.

Liveweight gain of HCP-HME steers was higher in Phase 1 (*P* < 0.001) and lower (*P* < 0.001) in Phase 2 compared with HCP-LME and LCP-LME steers, with no difference (*P* > 0.05) in LWG between HCP-LME and LCP-LME steers in either phase (Table 2). Holstein-Friesian steers had higher LWG than BX steers in Phase 1 (*P* < 0.05) and a tendency to higher LWG in Phase 2 (*P* = 0.08). There were significant interactions (*P* < 0.05) between nutritional treatment and genotype in both phases of the experiment. During Phase 1, HF steers consuming lucerne chaff *ad libitum* grew faster than BX steers (1.37 *vs* 1.17 kg/d; *P* = 0.04; not shown) allocated to the same nutritional treatment. The HF steers fed LCP-LME during Phase 1 showed a trend for greater LWG than HF steers fed HCP-LME during Phase 2 (*P* = 0.07). This was also the only group that showed higher LWG (1.5 *vs* 1.3 kg/d; *P* < 0.05) during Phase 2 when compared to HCP-HME steers during Phase 1. Steers fed ME restricted treatments (HCP-LME and LCP-LME) had lower feed conversion than HCP-HME steers during Phase 1 (*P* < 0.001). During Phase 2, the previously restricted steers converted DM into LW more efficiently than steers fed the HCP-HME nutritional treatment throughout the experiment (*P* < 0.001). The HF steers converted DM to LW more efficiently than BX steers in Phase 2 (*P* < 0.001) but no genotype differences in FCR were observed in Phase 1 (*P* > 0.05). The pattern of LWG during compensatory growth in Phase 2 followed closely DM intake, also reaching a peak approximately 60 d after the beginning of Phase 2 (Fig 2).

Steers fed the HCP-HME treatment had the highest HHG (*P* < 0.001), followed by HCP-LME and LCP-LME steers in Phase 1 (Table 2). A significant interaction (*P* < 0.05) between nutritional treatment and genotype existed in Phase 1 and the differences followed the same pattern as LWG. Holstein-Friesian steers fed HCP-HME had a higher rate of HHG than BX steers when fed the same nutritional treatment (*P* < 0.05). During Phase 2, HCP-LME and LCP-LME steers displayed skeletal catch-up growth and had a higher rate of HHG compared to HCP-HME steers (*P* < 0.05). The HHG of previously restricted steers (i.e. HCP-LME and LCP-LME) during Phase 2 was not significantly different to that of HCP-HME steers during Phase 1 (*P* > 0.05). The HF steers had a higher rate of HHG when compared with BX steers in both phases of the experiment (*P* < 0.05).

**Fig 3:**
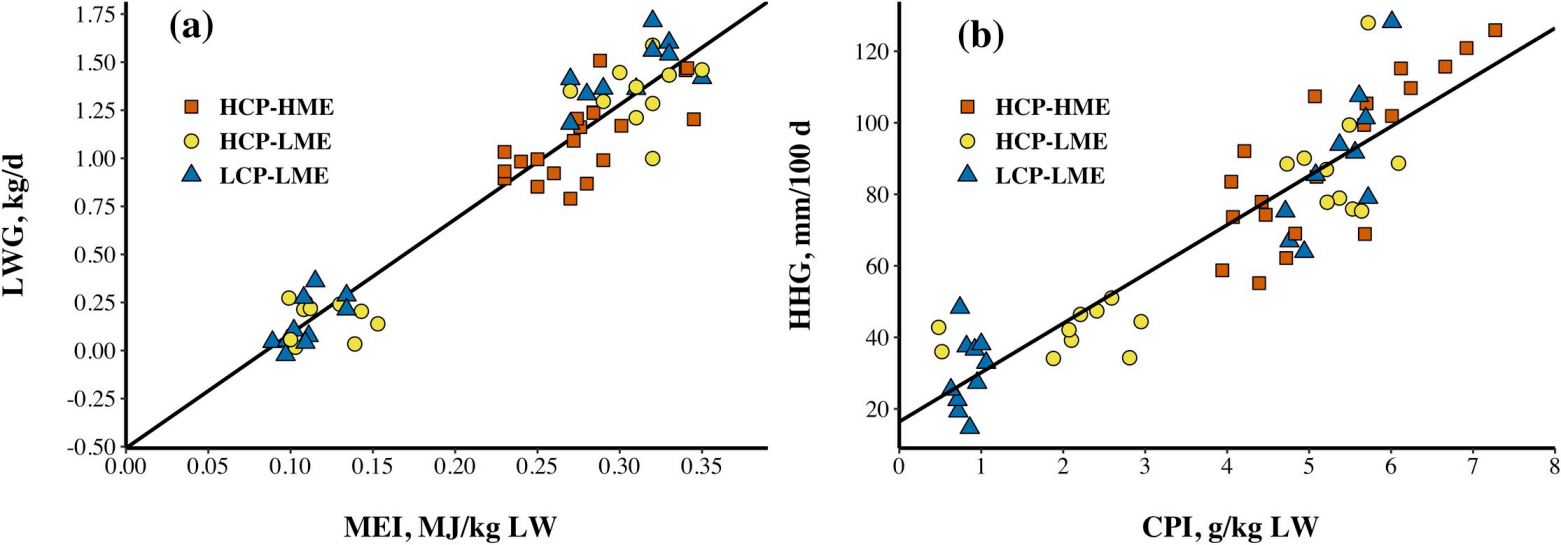
Effect of metabolizable energy (ME) intake (MEI) on liveweight gain (LWG; a; Y = -0.507 +5.95X, R^2^ = 0.92, RSE = 0.16, *P* < 0.001) and crude protein (CP) intake (CPI) on hip height gain (HHG; b; Y = 16.41 + 13.75X, R^2^ = 0.82, RSE = 13, *P* < 0.001) of steers fed high CP content and high ME intake (HCP-HME), high CP content and low ME intake (HCP-LME) and low CP content and low ME intake (LCP-LME). Each symbol represents the mean for an individual steer from Phase 1 and Phase 2 of the experiment.

The stepwise linear regressions analysis (Fig 3) of LWG and HHG on ME and CP intake showed ME intake to be the best predictor of LWG (R^2^ = 0.92) while HHG was best explained by CP intake (R^2^ = 0.82). The genotype effect and its interaction were not significant in any model therefore it was excluded from the final version.

### Plasma metabolites and hormones

The concentration of glucose and Ca in plasma were both higher (*P* < 0.001) in steers consuming HCP-HME treatment during Phase 1 (Table 3). The concentration of PUN was higher (*P* < 0.001) in the plasma of steers fed diets with a high CP content regardless of ME intake. Nutritional treatment had no effect (*P* > 0.05) on the concentration of total protein, NEFA or inorganic P in the plasma during Phase 1 of the experiment. The concentration of NEFA and PUN was higher (*P* < 0.01) in the plasma of BX steers compared with HF steers at the end of both phases of the experiment. In addition, the concentration of total protein showed a tendency (*P* = 0.06) to be greater in the plasma of HF than BX cattle in both phases. There were no significant (*P* > 0.05) differences in any of the metabolites analyzed during Phase 2 of the experiment when steers were consuming the same HCP-HME treatment.

**Table 3:** The concentration of metabolites and hormones in the plasma of Holstein-Friesian (HF) and Brahman crossbred (BX) steers fed different nutritional treatments (Phase 1) and undergoing re-alimentation (Phase 2).

Item^1^^,^^2^	Nutrition (N)			Genotype (G)		SEM	*P*-Value		
	HCP-HME	HCP-LME	LCP-LME	HF	BX		N	G	N x G
*Phase 1*									
Glucose, mmol/L	5.2^b^	3.9^a^	3.7^a^	4.2	4.3	0.14	<0.001	0.82	0.36
Total Protein, g/L	65.7	66.1	64.7	67	64	1.8	0.62	0.06	0.46
NEFA, mEq/L	0.2	0.16	0.11	0.1	0.22	0.04	0.24	0.001	0.45
PUN, mmol/L	7.1 ^b^	7.5^b^	1.2^a^	4.8	5.8	0.4	<0.001	0.007	0.92
P, mmol/L	2.3	2.1	2.3	2.2	2.3	0.14	0.22	0.62	0.85
Ca, mmol/L	2.4^b^	2.1^a^	2.1^a^	2.2	2.2	0.04	<0.001	0.90	0.28
Insulin, IU/mL	4.49^b^	1.68^a^	1.69^a^	2.57	2.67	0.71	<0.001	0.86	0.91
IGF-1, ng/mL	549.7^b^	146.6^a^	83.8^a^	239.2	280.9	78.7	<0.001	0.61	0.35
Leptin, ng/mL	4.70	4.01	3.23	3.46	4.49	0.80	0.22	0.10	0.31
T4, nmol/L	68.6^b^	69.7^b^	49.9^a^	59.5	65.9	7.38	0.01	0.28	0.01
T3, nmol/L	2.86^c^	2.19^b^	1.45^a^	2.32	2.01	0.23	<0.001	0.13	0.82
***Phase 2***									
Glucose, mmol/L	4.8	4.9	5	4.9	4.8	0.14	0.19	0.28	0.63
Total Protein, g/L	67.2	65	65.9	67.4	64.6	1.8	0.52	0.06	0.35
NEFA, mEq/L	0.14	0.16	0.13	0.09	0.19	0.04	0.74	0.007	0.35
PUN, mmol/L	7.7	7.8	7.4	7	8.3	0.3	0.64	<0.001	0.04
P, mmol/L	2.2	2.3	2.3	2.3	2.3	0.12	0.70	0.81	0.84
Ca, mmol/L	2	2.1	2.2	2.1	2	0.07	0.23	0.17	0.61
Insulin, IU/mL	5.22	4.46	4.28	5.29	4.01	1.75	0.72	0.21	0.40
IGF-1, ng/mL	571.6	403.7	372.5	481.0	417.5	117.7	0.32	0.12	0.19
Leptin, ng/mL	3.82	4.12	3.96	3.87	4.06	0.71	0.91	0.73	0.72
T4, nmol/L	69.6	56.5	70.5	73.1	57.9	10.9	0.14	0.35	0.31

^a-c^ Means within a row with different superscripts differ (*P* < 0.05).

^1^ Glucose, total protein, non-esterified fatty acids (NEFA), plasma urea nitrogen (PUN), inorganic phosphorus (P) and total calcium (Ca).

^2^ Insulin, insulin-like growth factor-1 (IGF-1), thyroxine (T4), leptin and of triiodothyronine (T3).

At the end of Phase 1 the concentration of insulin and IGF-1 were higher (*P* < 0.001) in the plasma of HCP-HME steers compared with steers allocated to the LME treatments (Table 3). The CP content of the diet had no effect on the concentration of insulin and IGF-1 in the plasma of LME steers. At the end of Phase 1, the concentration of T3 and T4 in plasma was higher (*P* < 0.001) in response to dietary CP content, while the concentration of T3 in plasma was also affected by ME intake. Genotype had no significant effect on the concentration of any hormones in plasma of steers at the end of Phase 1 or Phase 2. However, a significant genotype and nutritional treatment interaction effect (*P* < 0.05) on the concentration of T4 in the plasma was measured at the end of Phase 1, with HF steers allocated to the LCP-LME treatment having a significantly lower concentration than HF steers offered the other treatments (2.7 *vs* 5.0 nmol/L; *P* = 0.05; not shown) while the concentration of T4 was unaffected by nutritional treatment in BX steers (*P* > 0.05).

### Growth plate and trabecular bone histomorphometry

Reduced ME intake decreased the height of the HZ of the growth plate independent of diet CP content (Table 4). Conversely, height of the PZ was only decreased (*P* < 0.05) in HF steers fed ME restricted diets but not in BX steers (*P* < 0.05; not shown). The diameter of the THC showed an additive effect for CP content and ME intake (*P* < 0.001). There were more (*P* = 0.04) chondrocytes per column at the HZ of steers allocated to the HCP-HME than HCP-LME treatments but no differences (*P* < 0.05) were found between LME treatment steers. At the end of Phase 2, the PZ of steers fed LME treatments in Phase 1 was higher than HCP-HME steers (*P* = 0.01). In addition, there was a tendency (*P* = 0.07) for the HZ to be significantly thicker in the LME treatments. At all sampling points, PZ (*P* < 0.05) and HZ (*P* < 0.05) were thicker in HF steers than in BX steers. The number of HC per column (*P* < 0.05) and the diameter of THC (*P* < 0.001) were also higher in HF than BX steers but only at the first baseline sampling (d -10; not shown).

**Table 4:** Growth plate^1^ and trabecular bone histomorphometry^2^ of Holstein-Friesian (HF) and Brahman crossbred (BX) steers fed different nutritional treatments (Phase 1) and undergoing re-alimentation (Phase 2).

Item	Nutrition (N)^3^^,^^4^			Genotype (G)		SEM	*P*-Value		
	HCP-HME	HCP-LME	LCP-LME	HF	BX		N	G	N x G
*Phase 1*									
PZ, μm	310^b^	242^a^	259^a^	285	255	12.0	<0.001	0.03	<0.01
HZ, μm	181^b^	135^a^	125^a^	157	137	8.72	<0.001	0.01	0.16
n of HC, n/column	7.09^b^	5.65^a^	6.37^a^^b^	6.44	6.30	0.38	0.04	0.72	0.95
THC, μm	27.2^c^	22.9^b^	18.9^a^	23.2	22.8	1.43	<0.001	0.63	0.20
Bv/Tv, %	31.1^b^	21.7^a^	18.7^a^	25.9	22.9	1.89	<0.005	0.07	0.08
Tb.Sp, μm	399^b^	509^a^	525^a^	434	522	29.8	0.006	0.006	0.22
Tb.Th, μm	201^b^	146^a^	125^a^	156	159	9.48	<0.001	0.90	0.02
BS, mm	21.1^b^	18.6^a^^b^	17.8^a^	20.6	17.7	1.17	0.01	0.004	0.21
***Phase 2***									
PZ, μm	242^a^	306^b^	301^a^^b^	319	249	12.9	0.01	<0.005	0.09
HZ, μm	151	182	181	186	157	16.1	0.07	0.05	0.52
n of HC, n/column	6.04	6.04	6.45	5.88	6.47	0.50	0.51	0.15	0.67
THC, μm	25.1	23.7	23.4	25.1	23.0	2.03	0.57	0.20	0.80
Bv/Tv, %	27.7	27.0	27.0	26.0	28.5	1.43	0.90	0.11	0.77
Tb.Sp, μm	415	421	453	408	455	24.6	0.43	0.11	0.19
Tb.Th, μm	184	177	176	169	189	8.84	0.74	0.04	0.18
BS, mm	21.1	20.9	20.1	20.8	20.6	1.27	0.68	0.81	0.38

^a-c^ Means within a row with different superscripts differ (*P*<0.05).

^1^ Height of proliferative zone (PZ), height of hypertrophic zone (HZ), number of hypertrophic chondrocytes (HC) per column (n of HC), diameter of terminal HC (THC).

^2^ Bone volume (Bv/Tv), trabecular separation (Tb.Sp), trabecular thickness (Tb.Th), bone surface (BS).

^3^ Data are least squares means with standard error of the mean (SEM).

^4^ Steers were fed nutritional treatments with a high crude protein content and high metabolizable energy intake (HCP-HME), high CP content and low ME intake (HCP-LME) and low CP content and low ME intake (LCP-LME) during Phase 1 and were offered the HCP-HME diet *ad libitum* during Phase 2.

**Fig 4:**
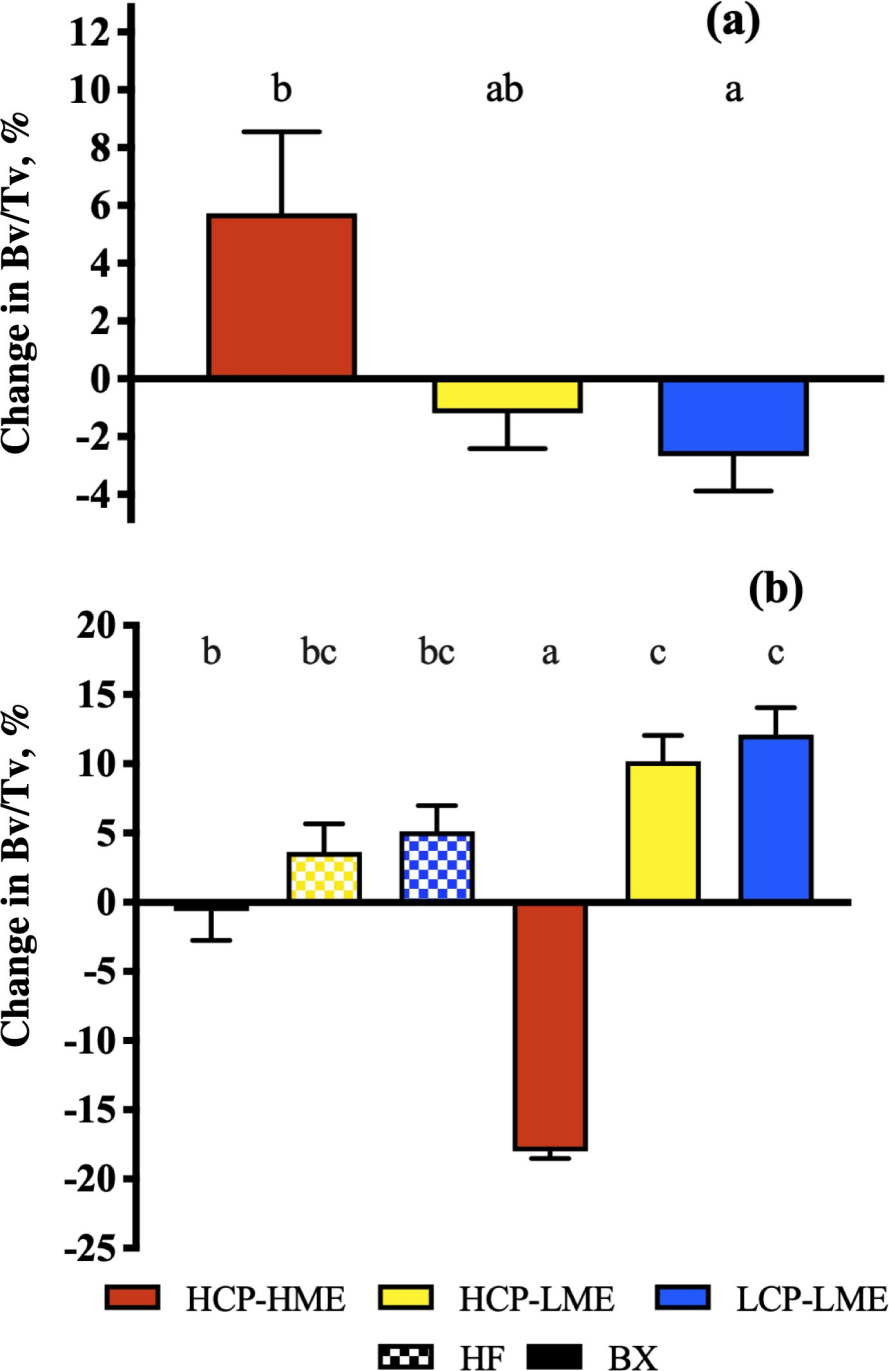
Change in trabecular bone volume (Bv/Tv) of tuber coxae samples collected from Holstein-Friesian (HF) and Brahman crossbred (BX) steers fed different nutritional treatments (Phase 1; a) and during re-alimentation (Phase 2; b). During Phase 1, the interaction between genotype and nutritional factors was not significant so the means of both genotypes were pooled by nutritional treatment. Data are expressed as mean ± SEM. Means with different letters are significantly different (Tukey, *P* < 0.05).

A significant difference between steers allocated to the different nutritional treatments was found in bone volume prior to the introduction of the treatments (*P* < 0.01; not shown). Trabecular bone of HCP-HME steers had higher volume (*P* < 0.005), surface (*P* = 0.01) and also smaller separation (*P* < 0.01) of trabecular bone at the end of Phase 1 compared with steers with restricted ME intake (Table 4). Only BX steers showed a statistically significant reduction (*P* < 0.05) in trabecular thickness due to reduced ME intake but there was no effect (*P* > 0.05) of CP content and hence intake during ME restriction. The LCP-LME steers had a significant loss of trabecular bone during Phase 1 compared to HCP-HME steers (*P* = 0.03; Fig 4). Prior to the start of nutritional treatments, HF steers had significantly higher bone volume (*P* = 0.03) and trabecular thickness (*P* = 0.01) than BX steers (not shown). In addition, at the end of Phase 1 BX steers had a more trabecular bone separation (*P* = 0.006) and smaller bone surface (*P* = 0.004) than HF steers. The differences in trabecular thickness, trabecular separation, bone surface and volume at the end of Phase 1 were no longer evident at the end of Phase 2. There was a significant interaction (*P* < 0.001) of genotype and nutritional treatment on the change in trabecular bone volume during Phase 2 (Fig 4B). The BX steers fed LME treatments during Phase 1 had an increase in bone volume when fed HCP-HME *ad libitum*. Trabecular separation was larger and bone surface smaller in BX than in HF steers at the end of Phase 1 but trabecular thickness was higher in BX steers (*P* = 0.04) at the end of Phase 2.

### Bone markers

During Phase 1, the concentration of BALP in plasma increased markedly in HCP-HME steers compared to steers consuming low energy treatments (i.e. HCP-LME and LCP-LME; *P* < 0.001; Table 5). For HCP-HME steers, the concentration of tDPD was significantly higher in the plasma of HCP-LME steers but not LCP-LME steers (*P* > 0.05). Further, a significant interaction (*P* = 0.04) between nutritional treatment and genotype was observed for the concentration of tDPD in plasma. The concentration of tDPD in plasma was lower in HF HCP-LME compared to HF HCP-HME steers (*P* = 0.01), at the end of Phase 1 but not in BX steers. The LCP-LME steers had a significantly (*P* < 0.05) higher concentration of PYD in plasma than HCP-LME steers but HCP-HME steers were not different to either other group. At the end of Phase 2 (d 203) HF steers had higher concentration of PYD (*P* = 0.02) and OCN (*P* < 0.01) and a lower concentration of tDPD (*P* < 0.01) in plasma than BX steers.

**Table 5:** The concentration of bone-specific alkaline phosphatase (BALP), osteocalcin (OCN), total deoxypyridinoline (tDPD) and pyridinoline (PYD) in the plasma of Holstein-Friesian (HF) and Brahman crossbred (BX) steers fed different nutritional treatments (Phase 1) and undergoing re-alimentation (Phase 2).

	Nutrition (N)^1^^,^^2^			Genotype (G)		SEM	*P*-Value		
Item	HCP-HME	HCP-LME	LCP-LME	HF	BX		N	G	N x G
*Phase 1*									
BALP, U/L	80.7^b^	21.5^a^	20.4^a^	40.9	40.3	9.3	<0.001	0.48	0.58
OCN, ng/mL	98.5	90.0	100.0	89.2	103.1	14.8	0.56	0.38	0.46
tDPD, nmol/L	8.41^b^	7.53^b^	7.87^a^^b^	7.86	8.01	0.27	0.04	0.60	0.04
PYD, nmol/L	3.24^a^^b^	2.86^a^	3.98^b^	3.67	3.05	0.26	0.02	0.05	0.07
***Phase 2***									
BALP, U/L	72.4	79.1	100.7	84.7	83.4	10.7	0.17	0.53	0.02
OCN, ng/mL	96.3	127.3	106.4	128.6	91.3	10.9	0.13	<0.01	0.59
tDPD, nmol/L	8.44	8.63	8.56	7.79	9.30	0.46	0.95	<0.01	0.36
PYD, nmol/L	2.49	2.63	2.13	3.31	1.53	0.34	0.23	<0.001	0.55

^a-c^ Means within a row with different superscripts differ (*P* < 0.05).

^1^ Data are least squares means with standard error of the mean (SEM).

^2^ Steers were fed nutritional treatments with a high crude protein content and high metabolizable energy intake (HCP-HME), high CP content and low ME intake (HCP-LME) and low CP content and low ME intake (LCP-LME) during Phase 1 and were offered the HCP-HME diet *ad libitum* during Phase 2.

Correlation analysis between bone marker concentration and trabecular bone histomorphometric parameters showed a significant positive correlation between the concentration of BALP in plasma and bone volume (r = 0.49, *P* < 0.001), trabecular thickness (r = 0.47, *P* < 0.001) and bone surface (r = 0.29, *P* < 0.05) and a negative correlation with trabecular separation (r = -0.31, *P* < 0.05). In addition, the concentration of PYD in plasma was negatively correlated with bone volume (r = -0.30, *P* < 0.05) and trabecular thickness (r = -0.36, *P* < 0.01). No other significant correlations existed between the concentration of circulating bone markers and bone histomorphometric parameters.

## Discussion

Skeletal growth and size are the main determinants of muscle mass and indirectly affect the composition of growth [3, 35, 36]. The objective of the current experiment was to increase skeletal growth, during energy restriction, through a higher CP intake leading to a deviation from the normal weight-for-height relationship. A greater deviation from the normal weight-for-height relationship would then lead to a greater LWG during re-alimentation (i.e. LW compensatory growth). In this work, we demonstrated that higher CP content and intake did stimulate bone growth during energy restriction, however this effect was mild and did not translate into greater LW compensatory growth. Higher CP intake appears to slightly compensate for the effect of energy restriction and this could be observed in HHG, differences in the diameter of terminal hypertrophic chondrocytes, bone surface, change in bone volume and the concentration of PYD in the plasma. Energy restriction played a major role in slowing the pace of endochondral ossification which led to skeletal catch-up growth (i.e. accelerated skeletal growth returning to a normal height-for-age) during re-alimentation (Phase 2). Holstein-Friesian steers had consistently greater thickness of the proliferative and hypertrophic zone of the growth plate and this was associated with higher ME intake and LWG than BX steers when offered a diet with high ME and CP content *ad libitum*. All steers exposed to nutritional restriction during Phase 1 exhibited compensatory and catch-up growth (i.e. LWG and HHG respectively) during Phase 2 when compared to steers that were unrestricted during Phase 1 of the experiment.

This study is not however free of limitations and we would like to discuss some of these. Both sex and castration affect circulating hormonal concentration and could potentially affect the physiological effects observed in this work. For instance, non-castrate cattle tend to have higher concentration of IGF-1 and testosterone in the plasma [37, 38], however the effect of nutritional restriction over different cattle categories (i.e. castrate male, intact male and females) seems to lead to similar changes in the endocrine system despite differences in overall values of hormonal concentration [11, 39–43]. We are unaware of any work which have applied nutritional restriction to cattle of different categories (i.e. castrate male, intact male and females) as a mean to assess these possible differences. In our research we used Brahman crossbred cattle which were 5/8 *Bos indicus* and 3/8 *Bos taurus* as well as Holstein-Friesian which are pure *Bos taurus*, therefore the differences in physiological response between genotypes should not be directly associated with differences between species (i.e. *Bos indicus* and *Bos taurus*). However, some of the differences we observed in Brahman crossbred steers (e.g. rumen ammonia) have been previously associated with *Bos indicus* animals [44]. Lastly, we do acknowledge that the frequency of bone and blood sampling could be increased to provide a more in depth understanding of changes across time. However, in this study we adopted a novel sampling technique in order to extract a bone specimen from the tuber coxae bone. Such a technique allowed us to perform bone histomorphometry analysis without slaughtering the animals but we were unsure about the effect of repeated sampling on the same site could have on the quality of these samples. Therefore, we opted to collect the minimum number of samples needed to test our initial hypothesis. We then aimed to combine these bone sampling dates with blood samples in order to provide an overview of the endocrine responses at the same point in time. The physiological changes and implications of the findings of this work are discussed.

### Nutritional parameters

The type of nutrient restriction (i.e. protein, energy or minerals) has been assumed to be one of the factors affecting compensatory growth [45]. Overall, the nutritional treatment design of this experiment was successful in imposing a clear separation between high and low CP diets during energy restriction. This distinction was confirmed not only by the differences in ME and CP intake and the DOM:CP but also by the glucose and PUN concentration in the plasma. The EMCP is a measure of the protein:energy ratio of absorbed substrates and clearly showed a much high ratio for the high CP rations. Moreover, the similarity in the concentration of Ca and P in the plasma of steers fed the ME restricted treatments (i.e. HCP-LME and LCP-LME) indicates that the nutritional effects on production and bone metabolism were independent of any mineral limitation.

Steers consuming high CP content treatments (i.e. HCP-HME and HCP-LME) had greater concentration of NH_3_H in the rumen than cattle consuming the low CP content regardless of genotype. However, BX steers had a substantially greater concentration (83.3 *vs* 16.2 mg/L) of NH_3_N than HF steers when both genotypes were consuming Mitchell grass hay (i.e. LCP-LME). Similarly, Hunter and Siebert [44] observed that Brahman steers fed spear grass (38 g CP/kg DM) had a greater concentration of NH_3_N in the rumen (40 *vs* 16 mg/L) than Hereford steers, but this genotype difference disappeared when a urea and sulphur supplement (30 g N/kg DOM) was offered.

Plasma urea nitrogen concentration did not reflect the concentration of NH_3_H in the rumen, instead it was more related to the relationship between CP to ME content in the diet. The DOM:CP of steers fed HCP-HME and HCP-LME in the present experiment was 3.3 and 3.5 respectively, while the PUN concentration was 7.1 and 7.5 mmol/L. Cattle fed LCP-LME had a much greater DOM:CP (12.1) and lower PUN concentration (1.2 mmol/L). In addition, it has been suggested that a DOM:CP of approximately 7:1 and greater corresponds to a limitation of rumen degradable protein and hence a response to N supplementation is expected (Moore et al., 1995; Moore et al., 1999). The HCP-HME and HCP-LME diets provided a much greater metabolizable protein:ME and a greater CP intake than the LCP-LME diet. This approach allows ME and CP effects to be separated for their influence on LWG, skeletal growth and the concentration of various hormones.

### Feed intake and growth of steers during re-alimentation

Energy-restricted steers had greater DM intake and LWG during re-alimentation when compared to steers that had *ad libitum* access to lucerne chaff during the entire experimental period. The pattern of DM intake during re-alimentation was strikingly similar between the previously restricted groups of steers. Steers fed restricted treatments during Phase 1 had increased DM intake and LWG over Phase 2 reaching a peak approximately 60 d after the start of the re-alimentation period. Interestingly, HF steers fed LCP-LME in Phase 1 showed a transitory greater LWG than HF steers fed HCP-LME during re-alimentation, without any increase in DM intake. In addition, this group of steers (i.e. HF steers fed LCP-LME) were the only restricted treatment group which showed greater LWG during LW compensatory growth (i.e. during Phase 2) than unrestricted HF steers fed high CP and ME at an approximately equivalent LW (during Phase 1).

The effect of nutritional restriction and re-alimentation on organs and digestive tract size was not evaluated in the present experiment. However, studies which have analysed these parameters indicate that during nutritional restriction there is a significant reduction in organ mass (e.g. liver and spleen) and digestive tract (e.g. stomach and intestine) [10, 46, 47]. During re-alimentation, Yambayamba et al. [10] observed an overcompensation of liver, spleen and stomach between 50 and 78 d after the start of re-alimentation. Despite the fact that the gastrointestinal tract and liver contribute less than 10% of the liveweight [48], they represent a high proportion or energy requirement. Moreover, the size of these organs is related to the capacity of the animal to process food and synthesize protein and may be the restricting factor limiting increase in voluntary food intake at early stages of compensatory growth. This hypothesis is in agreement with Ryan et al. [49] who suggested that only after the liver and digestive tract are replenished that compensating animals would be able to increase their feed intake above that of non-restricted cohorts. This may explain the steady increase in pattern of DMI and LWG observed at the early stages of the re-alimentation phase in the present study and elsewhere [50]. In this scenario, the decrease in organs and muscles during nutritional restriction would represent a potential for protein deposition and lead to stimulus of voluntary intake [51–55].

The stepwise linear regressions analysis of LWG and HHG on ME and CP intake showed ME intake to be the best predictor of LWG (R^2^ = 0.92) while HHG was best explained by CP intake (R^2^ = 0.82). These responses were independent of genotype. The increased DM intake during the *ad libitum* phase seems to be the main mechanism driving higher growth rates following ME and CP restriction. A similar conclusion was also reached by other researchers investigating the compensatory growth phenomenon but there is currently limited information available to explain the long-term control of voluntary intake following periods of feed restriction [41, 49].

### Skeletal growth

Skeletal elongation was assessed directly by HH measurements across time and indirectly by histomorphometric assessments of the tuber coxae growth plate. Overall, the results obtained by both methods agreed on the differences observed in skeletal growth caused by nutritional and genotype treatments. Independent of CP content and intake, ME restriction severely reduced endochondral ossification. This impact can be observed by reductions in the thickness of the proliferative and hypertrophic zones as well as in the number and diameter of terminal hypertrophic chondrocytes. However, steers fed a high CP content diet during ME restriction had a significantly greater diameter of terminal hypertrophic chondrocytes when compared to counterparts fed the low CP content diet. This result may help explain the greater HHG observed in these steers. Wilsman et al. [56] suggested that longitudinal growth is a function of the number of cells produced through proliferation multiplied by the volume of terminal hypertrophic chondrocytes. High correlations have been reported between the volume of hypertrophic cells and bone elongation rate in rats (r = 0.98) and pigs (r = 0.83) [57].

During re-alimentation (i.e. Phase 2), previously restricted steers showed a thicker proliferative zone as well as a tendency (*P* = 0.07) for a thicker hypertrophic zone than previously unrestricted steers. These results also agree with the differences in skeletal growth measured by HHG in these steers. In addition, these results support that faster bone elongation after a period of nutritional restriction (i.e. catch-up growth) in cattle may be explained by the growth plate delayed senescence hypothesis [14, 15, 17]. This hypothesis assumes that growth plate senescence depends on the replicative history of chondrocytes at the growth plate resting zone. Therefore, once growth limiting conditions are resolved, growth plates of restricted animals are more “physiologically immature” than unrestricted cohorts at the same age. It is also important to point out that differences observed in this work on growth plate morphology during re-alimentation were not related to any changes in plasma concentration of endocrine or metabolite parameters, suggesting that apart from the well-known endocrine factors that regulate linear growth [58] there are also local controlling mechanisms intrinsic to the growth plate, which was previously suggested by Lui and Baron [16].

In relation to the effect of protein intake on HHG, the results observed in the present investigation are in agreement with the work reported by Frandsen et al. [20] when working with rodents. The results show a more severe retardation in terms of skeletal growth, as measured by trunk and tail length, tibial length, tibial width through the proximal epiphysis and the middle portion of the diaphysis, and width of the tibial proximal epiphyseal cartilage plate in rodents fed the low protein diets than pair-fed groups.

Over both experimental phases (i.e. Phase 1 and 2), HF had thicker proliferative and hypertrophic zones when compared to BX steers. Interestingly, restricted ME intake reduced the height of hypertrophic zone in both genotypes but only BX steers showed reductions on the proliferative zone.

### Trabecular bone turnover and bone marker concentration

Studies with other species have shown that energy and protein restriction affect bone turnover (i.e. bone formation and resorption) leading to an osteoporotic state [59, 60]. Taken all together the results presented here indicate that trabecular bone loss at the tuber coxae is a result of a greater reduction in bone formation than an increase in bone resorption. Restricted ME intake led to severe reductions in the concentration of BAP, bone volume, trabecular thickness and bone surface as well as increase in trabecular separation. However, only steers fed low CP content diets during ME restriction showed significant reductions in bone surface and change in bone volume when compared to steers fed the HCP-HME diet. These results were also associated with a significantly lower concentration of PYD in the plasma of HCP-LME when compared to LCP-LME steers which may indicate that higher CP intake during ME restriction slightly decreased bone resorption. This suggestion is in agreement with Mardon et al. [61] who observed significant bone sparing assessed through bone mineral density in growing rats when energy restriction was associated with adequate protein intake but no differences in plasma IGF-1 concentration.

The changes in trabecular structure caused by bone loss during ME restriction were completely reversed by the end of the re-alimentation period (i.e. Phase 2). The change in bone volume shows that previously restricted steers had significantly higher net bone balance during re-alimentation. Pando et al. [6] showed that after only one day of re-alimentation there was a significant increase in collagen fibre deposition in the trabecular bone of previously caloric restricted rats. This was accompanied by a rise in BALP and IGF-1 to the same concentration as a control group. In a similar manner, we also did not observe any differences in bone markers or hormone concentration between the different nutritional treatment groups at the end of Phase 2.

Some earlier publications which have reported the use of bone biomarkers in bovines have been mainly focused on dairy cattle [62–64] and beef breeder cows [65] during pregnancy and lactation. Apart from the obvious differences in physiology between reproductive and lactating cows to growing steers, it appears that this is the first study that aligns bone marker concentration with direct morphological measures of bone in cattle. The examination of the relationship between the concentration of such markers and changes in bone structure is essential for the validation of this technique [66].

The current study showed that independent of CP content and intake, cattle had a similar concentration of BALP in the plasma during energy restriction. However, steers fed a high CP treatment had significantly lower concentration of PYD in the plasma than unrestricted steers which indicates lower bone resorption activity. In HF cows, studies demonstrated that BALP concentration decreases with age [67], parity [68] and as cows transitioned from parturition to lactation [63] which is similar to changes reported in humans [69]. Delmas et al. [70] observed a significant positive correlation between bone resorption measures and concentration of urinary PYD (r = 0.35, *P* < 0.05). In HF cows the concentration of PYD in serum and urine peaked soon after calving and decreased again as lactation progressed [62, 64]. Also, a lower serum PYD concentration was observed in early lactation of HF cows fed a low calcium diet (4.6 g Ca/kg DM) when compared to a group fed a high calcium diet (6.4 g Ca/kg DM) [71]. Overall, these observations agree with the changes observed in the current study. The rapid shift in energy requirements of high producing dairy cows around parturition leads to an increase in bone mobilization during negative energy balance and these changes are associated with changes in BALP and PYD concentration [63, 64, 72].

### Endocrine responses

The higher HHG in cattle fed HCP-LME was accompanied by a significant increase in the concentration of T3 in plasma. Thyroid hormones are directly involved with body growth, skeletal development, energy metabolism and temperature homeostasis [73–75]. Thyroxin is the most abundant in the bloodstream but T3 is the most active form of the thyroid hormones. It is not known if the higher T3 concentration in the plasma of steers consuming high CP diets could exert a direct stimulatory effect on chondrocyte hypertrophy and consequently growth rates. Hypothyroidism in humans is known to reduce growth rates and skeletal maturation [76].

Interestingly, ME restriction did not affect the concentration of T4 in the plasma of BX steers and it only decreased the concentration in the plasma of HF steers when it was coupled with a lower CP content in the diet. This may indicate that energy and protein balance of the diet affects the deiodination process. In addition, it also indicates a different physiological response between genotypes. Investigations in caloric restricted rats fed low protein diets show an increase in the T3 concentration above controls [77–80] which is not explained by enhanced conversion of T4 to T3 nor by the maximum binding capacity of T3 in the liver or binding affinity with receptors [81]. In cattle, there is no information regarding the effect of a low or high protein diet during energy restriction on the concentration of thyroid hormones in the plasma.

The effects of growth hormone on the growth plate are both direct where growth hormone acts on locally recruiting chondrocytes in the resting zone to differentiate into proliferative chondrocytes, and indirect where growth hormone promotes local production of IGF-1 which then stimulates the proliferation of proliferative chondrocytes [82]. The somatotrophic axis is sensitive to nutrient supply, with protein and energy intake modulating the concentration and action of growth hormone and IGF-1. Restricted energy intake reduced linear growth in cattle which was associated with reduced concentration of IGF-1 in plasma [11, 39]. Elsasser et al. [19] observed a linear increase in the plasma concentration of IGF-1 with increasing CP content of a high energy content diet (12.3 MJ/kg DM). However, steers fed a lower energy content diet (8.2 MJ/kg DM) did not show any increase in IGF-1 concentration when fed a 110 or 140 g CP/kg DM diet but IGF-1 concentration was greater than in steers fed an 80 g CP/ kg DM diet. Similarly, no significant differences were observed in the present study when comparing IGF-1 concentration in the plasma of steers fed high (199 g CP/ kg DM) and low CP (38 g CP/ kg DM) content diets during energy restriction. This could suggest that during energy restriction, plasma IGF-1 concentration does not have an association with the rate of skeletal growth. Alternatively, the stimulatory effect of a higher CP diet on endochondral ossification could be mediated by the autocrine-paracrine action of local IGF-1 production. This explanation is based on the observation that dietary CP intake stimulates non-hepatic IGF-1 production [83]. Moreover, in mice with a loss of IGF-1 production in chondrocytes and osteoblasts, changes in endochondral ossification occur without changes in the concentration of IGF-1 in plasma [84, 85].

## Conclusions

Higher CP content of the diet caused a significant but still biologically small stimulus on bone growth and reduction of bone loss in steers under restricted ME intake. The greater gain in HH observed for the HCP-LME group during energy restriction was associated with greater T3 concentration in the plasma and larger terminal hypertrophic chondrocytes when compared to LCP-LME. However, it is unknown if the greater T3 concentration in the plasma could be the cause of this faster skeleton growth rate.

The differences observed between the Brahman crossbred and Holstein-Friesian genotypes suggest a different physiological response between the two genotypes when submitted to diets that result in altered ME and CP intake. Contrary to the initial hypothesis, HF steers fed the LCP-LME diet were the only steers to demonstrate greater LWG during compensatory growth compared with unrestricted HF steers. This response was associated with a lower concentration of T4 in the plasma during nutritional restriction and no differences in DM intake during recovery which may suggest a further reduction in basal metabolic rate and maintenance requirements when compared to steers with a higher CP intake.

The greater skeletal elongation rate during re-alimentation as well as changes observed at the growth plate level (e.g. proliferative and hypertrophic zone) suggests that skeletal catch-up growth in cattle can also be explained by the delayed senescence hypothesis. Steers which have experienced a period of restricted ME intake display greater skeletal growth than unrestricted steers at the same age (i.e. catch-up growth) once offered *ad libitum* access to a non-limiting diet. The concentration of BALP and PYD in plasma are proposed to be potential markers of bone formation and resorption, respectively. The rate of skeletal growth of cattle during catch-up growth is independent of CP content and intake during the restriction period. Therefore, increasing CP intake during ME restriction does not enhance LW compensatory nor skeletal catch-up growth in cattle.

## Supporting information

S1 Appendix Colorimetric, radioimmuno and enzyme linked assays. (DOCX)

S1 File (XLSX)

## References

[1] Coutinho EL, Gomes ARS, França CN, Oishi J, Salvini TF. Effect of passive stretching on the immobilized soleus muscle fiber morphology. Brazilian Journal of Medical and Biological Research. 2004;37:1853–1861. 10.1590/s0100-879x2004001200011 15558192

[2] Williams PE, Goldspink G. The effect of immobilization on the longitudinal growth of striated muscle fibres. Journal of Anatomy. 1973;116(Pt 1):45–55. PMC1271549. 4798240 PMC1271549

[3] Young M, Sykes A. Bone growth and muscularity. Proceedings of the New Zealand Society of Animal Production 1987, Vol. 47: 73–75.

[4] Huxley JS, Teissier G. Terminology of relative growth. Nature. 1936;137(3471):780–781.

[5] Ashworth A. Growth rates in children recovering from protein-calorie malnutrition. British journal of nutrition. 1969;23(04):835–845. 10.1079/bjn19690094 5357048

[6] Pando R, Masarwi M, Shtaif B, Idelevich A, Monsonego-Ornan E, Shahar R, et al. Bone quality is affected by food restriction and by nutrition-induced catch-up growth. Journal of Endocrinology. 2014;223(3):227–239. 10.1530/JOE-14-0486 25248555

[7] Damen GM, Boersma B, Wit JM, Heymans HSA. Catch-up Growth in 60 Children with Celiac Disease. Journal of Pediatric Gastroenterology and Nutrition. 1994;19(4):394–400. 10.1097/00005176-199411000-00005 . 7876992

[8] Gonzalez-Bulnes A, Ovilo C, Lopez-Bote CJ, Astiz S, Ayuso M, Perez-Solana ML, et al. Gender-specific early postnatal catch-up growth after intrauterine growth retardation by food restriction in swine with obesity/leptin resistance. Reproduction. 2012;144(2):269–278. 10.1530/REP-12-0105 22692087

[9] Sliwa E, Dobrowolski P, Piersiak T. Bone development of suckling piglets after prenatal, neonatal or perinatal treatment with dexamethasone. Journal of Animal Physiology and Animal Nutrition. 2010;94(3):293–306. 10.1111/j.1439-0396.2008.00909.x 19663986

[10] Yambayamba ESK, Price MA, Jones SDM. Compensatory growth of carcass tissues and visceral organs in beef heifers. Livestock Production Science. 1996;46(1):19–32. 10.1016/0301-6226(96)00014-0.

[11] Blum JW, Schnyder W, Kunz PL, Blom AK, Bickel H, Schurch A. Reduced and compensatory growth—endocrine and metabolic changes during food restriction and refeeding in steers. Journal of Nutrition. 1985;115(4):417–424. WOS:A1985AFP7000001. 10.1093/jn/115.4.417 3884752

[12] Ryan WJ. Compensatory growth in cattle and sheep. Nutrition Abstracts and Reviews Series B, Livestock Feeds and Feeding 1990. p. 653–664.

[13] Jobling M. Are compensatory growth and catch-up growth two sides of the same coin? Aquaculture International. 2010;18(4):501–510. 10.1007/s10499-009-9260-8

[14] Baron J, Klein KO, Colli MJ, Yanovski JA, Novosad JA, Bacher JD, et al. Catch-up growth after glucocorticoid excess: A mechanism intrinsic to the growth plate. Endocrinology. 1994;135(4):1367–1371. 10.1210/endo.135.4.7925098 7925098

[15] Gafni RI, Weise M, Robrecht DT, Meyers JL, Barnes KM, De-Levi S, et al. Catch-up growth is associated with delayed senescence of the growth plate in rabbits. Pediatric research. 2001;50(5):618–623. 10.1203/00006450-200111000-00014 11641457

[16] Lui JC, Baron J. Mechanisms limiting body growth in mammals. Endocrine reviews. 2011;32(3):422–440. 10.1210/er.2011-0001 21441345 PMC3365796

[17] Gat-Yablonski G, Shtaif B, Abraham E, Phillip M. Nutrition-induced catch-up growth at the growth plate. J Pediatr Endocrinol Metab. 2008;21(9):879–893. Epub 2008/10/18. 10.1515/jpem.2008.21.9.879 . 18924581

[18] Mohan S, Kesavan C. Role of Insulin-like Growth Factor-1 in the Regulation of Skeletal Growth. Current Osteoporosis Reports. 2012;10(2):178–186. 10.1007/s11914-012-0100-9 22544603

[19] Elsasser TH, Rumsey TS, Hammond AC. Influence of Diet on Basal and Growth Hormone-Stimulated Plasma Concentrations of IGF-I in Beef Cattle1. Journal of Animal Science. 1989;67(1):128–141. 10.2527/jas1989.671128x 2925537

[20] Frandsen AM, Nelson MM, Sulon E, Becks H, Evans HM. The effects of various levels of dietary protein on skeletal growth and endochondral ossification in young rats. The Anatomical Record. 1954;119(2):247–265. 10.1002/ar.1091190208 13189142

[21] Bourrin S, Toromanoff A, Ammann P, Bonjour JP, Rizzoli R. Dietary Protein Deficiency Induces Osteoporosis in Aged Male Rats. Journal of Bone and Mineral Research. 2000;15(8):1555–1563. 10.1359/jbmr.2000.15.8.1555 10934654

[22] Yayha Z, Millward DJ. Dietary protein and the regulation of long-bone and muscle growth in the rat. Clinical Science. 1994;87(2):213–224. 10.1042/cs0870213 7924167

[23] Freer M, Dove H, Nolan JV. Nutrient Requirements of Domesticated Ruminants: CSIRO Publishing; 2007.

[24] Van Soest PJ, Robertson JB, Lewis BA. Methods for Dietary Fiber, Neutral Detergent Fiber, and Nonstarch Polysaccharides in Relation to Animal Nutrition. Journal of Dairy Science. 1991;74(10):3583–3597. 10.3168/jds.S0022-0302(91)78551-2 1660498

[25] Balcells J, Guada JA, Peiró JM, Parker DS. Simultaneous determination of allantoin and oxypurines in biological fluids by high-performance liquid chromatography. Journal of Chromatography B: Biomedical Sciences and Applications. 1992;575(1):153–157. 10.1016/0378-4347(92)80517-t 1517293

[26] Chen XB, Gomes M. Estimation of microbial protein supply to sheep and cattle based on urinary excretion of purine derivatives-an overview of the technical details: International Feed Resources Unit; 1995.

[27] Bowen MK, Poppi DP, McLennan SR, Doogan VJ. A comparison of the excretion rate of endogenous purine derivatives in the urine of Bos indicus and Bos taurus steers. Australian Journal of Agricultural Research. 2006;57(2):173–177. 10.1071/AR05182.

[28] Callis G, Sterchi D. Decalcification of Bone: Literature Review and Practical Study of Various Decalcifying Agents. Methods, and Their Effects on Bone Histology. Journal of Histotechnology. 1998;21(1):49–58.

[29] Schneider CA, Rasband WS, Eliceiri KW. NIH Image to ImageJ: 25 years of image analysis. Nat Meth. 2012;9(7):671–675. 10.1038/nmeth.2089 22930834 PMC5554542

[30] Doube M, Kłosowski MM, Arganda-Carreras I, Cordelières FP, Dougherty RP, Jackson JS, et al. BoneJ: Free and extensible bone image analysis in ImageJ. Bone. 2010;47(6):1076–1079. 10.1016/j.bone.2010.08.023 20817052 PMC3193171

[31] Dempster DW, Compston JE, Drezner MK, Glorieux FH, Kanis JA, Malluche H, et al. Standardized Nomenclature, Symbols, and Units for Bone Histomorphometry: A 2012 Update of the Report of the ASBMR Histomorphometry Nomenclature Committee. Journal of bone and mineral research: the official journal of the American Society for Bone and Mineral Research. 2013;28(1):2–17. 10.1002/jbmr.1805 PMC3672237. 23197339 PMC3672237

[32] R Core Team. R: A language and environment for statistical computing. 2013.

[33] Pinheiro J, Bates D, DebRoy S, Sarkar D. R Core Team (2014) nlme: linear and nonlinear mixed effects models. R package version 3.1–117. See http://CRANR-projectorg/package=nlme. 2014.

[34] Osborne JW. Improving your data transformations: Applying the Box-Cox transformation. Practical Assessment, Research & Evaluation. 2010;15(12):1–9.

[35] Holly R, Barnett J, Ashmore C, Taylor R, Mole P. Stretch-induced growth in chicken wing muscles: a new model of stretch hypertrophy. American Journal of Physiology-Cell Physiology. 1980;238(1):C62–C71. 10.1152/ajpcell.1980.238.1.C62 7356012

[36] Alway SE, Gonyea WJ, Davis ME. Muscle fiber formation and fiber hypertrophy during the onset of stretch-overload. American Journal of Physiology-Cell Physiology. 1990;259(1):C92–C102. 10.1152/ajpcell.1990.259.1.C92 . 2142581

[37] Hunt DW, Henricks DM, Skelley GC, Grimes LW. Use of trenbolone acetate and estradiol in intact and castrate male cattle: effects on growth, serum hormones, and carcass characteristics2. Journal of Animal Science. 1991;69(6):2452–2462. 10.2527/1991.6962452x 1885362

[38] Lee CY, Henricks DM, Skelley GC, Grimes LW. Growth and hormonal response of intact and castrate male cattle to trenbolone acetate and estradiol2. Journal of Animal Science. 1990;68(9):2682–2689. 10.2527/1990.6892682x 2211398

[39] Yambayamba ESK, Price MA, Foxcroft GR. Hormonal status, metabolic changes, and resting metabolic rate in beef heifers undergoing compensatory growth. Journal of Animal Science. 1996;74(1):57–69. WOS:A1996TY73800009. 10.2527/1996.74157x 8778113

[40] Hornick JL, Van Eenaeme C, Diez M, Minet V, Istasse L. Different periods of feed restriction before compensatory growth in Belgian Blue bulls: II. Plasma metabolites and hormones. Journal of Animal Science. 1998;76(1):260–271. 10.2527/1998.761260x 9464907

[41] Keogh K, Waters S, Kelly A, Kenny D. Feed restriction and subsequent realimentation in Holstein Friesian bulls: I. Effect on animal performance; muscle, fat, and linear body measurements; and slaughter characteristics. Journal of animal science. 2015;93(7):3578–3589. 10.2527/jas.2014-8470 26440026

[42] Ellenberger MA, Johnson DE, Carstens GE, Hossner KL, Holland MD, Nett TM, et al. Endocrine and Metabolic Changes during Altered Growth Rates in Beef Cattle. Journal of Animal Science. 1989;67(6):1446–1454. 10.2527/jas1989.6761446x 2670867

[43] Hayden JM, Williams JE, Collier RJ. Plasma growth hormone, insulin-like growth factor, insulin, and thyroid hormone association with body protein and fat accretion in steers undergoing compensatory gain after dietary energy restriction. Journal of Animal Science. 1993;71(12):3327–3338. 10.2527/1993.71123327x 8294284

[44] Hunter R, Siebert S. Utilization of low-quality roughage by Bos taurus and Bos indicus cattle. British Journal of Nutrition. 1985;53(03):637–648. 10.1079/bjn19850073 2998449

[45] Wilson PN, Osbourn DF. Compensatory growth after undernutrition in mammals and birds. Biological Reviews. 1960;35(3):324–361. 10.1111/j.1469-185x.1960.tb01327.x 13785698

[46] Drouillard JS, Klopfenstein TJ, Britton RA, Bauer ML, Gramlich SM, Wester TJ, et al. Growth, body composition, and visceral organ mass and metabolism in lambs during and after metabolizable protein or net energy restrictions. Journal of Animal Science. 1991;69(8):3357–3375. 10.2527/1991.6983357x 1894573

[47] Ryan W, Williams I, Moir R. Compensatory growth in sheep and cattle. II. Changes in body composition and tissue weights. Australian Journal of Agricultural Research. 1993;44(7):1623–1633. 10.1071/AR9931623.

[48] Ferrell CL. Contribution of visceral organs to animal energy expenditures. Journal of Animal Science. 1988;66(suppl_3):23–34. 10.1093/ansci/66.Supplement_3.23

[49] Ryan W, Williams I, Moir R. Compensatory growth in sheep and cattle. 1. Growth pattern and feed intake. Australian Journal of Agricultural Research. 1993;44(7):1609–1621. 10.1071/AR9931609.

[50] Hornick JL, Van Eenaeme C, Gerard O, Dufrasne I, Istasse L. Mechanisms of reduced and compensatory growth. Domest Anim Endocrinol. 2000;19(2):121–132. Epub 2000/10/12. 10.1016/s0739-7240(00)00072-2 . 11025191

[51] Radcliffe JD, Webster AJF. Regulation of food intake during growth in fatty and lean female Zucker rats given diets of different protein content. British Journal of Nutrition. 1976;36(3):457–469. Epub 03/09. 10.1079/BJN19760100 1009072

[52] Radcliffe JD, Webster AJF. The effect of varying the quality of dietary protein and energy on food intake and growth in the Zucker rat. British Journal of Nutrition. 1979;41(1):111–124. Epub 12/08. 10.1079/bjn19790018 420743

[53] Cooper SDB, Kyriazakis I, Oldham JD. The effect of late pregnancy on the diet selections made by ewes. Livestock Production Science. 1994;40(3):263–275. 10.1016/0301-6226(94)90094-9.

[54] Webster AJF. Energy partitioning, tissue growth and appetite control. Proceedings of the Nutrition Society. 1993;52(1):69–76. Epub 02/28. 10.1079/pns19930038 8493278

[55] Kyriazakis I, Oldham JD. Diet selection in sheep: the ability of growing lambs to select a diet that meets their crude protein (nitrogen × 6.25) requirements. British Journal of Nutrition. 1993;69(3):617–629. Epub 03/01. 10.1079/bjn19930064 8329339

[56] Wilsman NJ, Bernardini ES, Leiferman E, Noonan K, Farnum CE. Age and pattern of the onset of differential growth among growth plates in rats. Journal of Orthopaedic Research. 2008;26(11):1457–1465. 10.1002/jor.20547 18404738 PMC2954232

[57] Breur GJ, Vanenkevort BA, Farnum CE, Wilsman NJ. Linear relationship between the volume of hypertrophic chondrocytes and the rate of longitudinal bone growth in growth plates. Journal of Orthopaedic Research. 1991;9(3):348–359. 10.1002/jor.1100090306 2010838

[58] Nilsson O, Marino R, De Luca F, Phillip M, Baron J. Endocrine Regulation of the Growth Plate. Hormone Research in Paediatrics. 2005;64(4):157–165. 10.1159/000088791 16205094

[59] Bourrin S, Ammann P, Bonjour JP, Rizzoli R. Dietary Protein Restriction Lowers Plasma Insulin-Like Growth Factor I (IGF-I), Impairs Cortical Bone Formation, and Induces Osteoblastic Resistance to IGF-I in Adult Female Rats. Endocrinology. 2000;141(9):3149–3155. 10.1210/endo.141.9.7633 . 10965885

[60] Devlin MJ, Cloutier AM, Thomas NA, Panus DA, Lotinun S, Pinz I, et al. Caloric restriction leads to high marrow adiposity and low bone mass in growing mice. Journal of Bone and Mineral Research. 2010;25(9):2078–2088. 10.1002/jbmr.82 20229598 PMC3127399

[61] Mardon J, Trzeciakiewicz A, Habauzit V, Davicco M-J, Lebecque P, Mercier S, et al. Dietary Protein Supplementation Increases Peak Bone Mass Acquisition in Energy-Restricted Growing Rats. Pediatric research. 2009;66(5):513–518. RS_600313998513ergyrestrictedgrowingrats. 10.1203/PDR.0b013e3181b9b4bb 19668107

[62] Liesegang A, Sassi ML, Risteli J, Eicher R, Wanner M, Riond JL. Comparison of Bone Resorption Markers During Hypocalcemia in Dairy Cows1,2. Journal of Dairy Science. 1998;81(10):2614–2622. 10.3168/jds.S0022-0302(98)75819-9 9812267

[63] Kim D, Yamagishi N, Ueki A, Miura M, Saito F, Sato S, et al. Changes in Plasma Bone Metabolic Markers in Periparturient Dairy Cows. Journal of Veterinary Medical Science. 2010;72(6):773–776. 10.1292/jvms.09-0409 20086325

[64] Elizondo Salazar JA, Ferguson JD, Beegle DB, Remsburg DW, Wu Z. Body phosphorus mobilization and deposition during lactation in dairy cows. Journal of Animal Physiology and Animal Nutrition. 2013;97(3):502–514. 10.1111/j.1439-0396.2012.01291.x 22452565

[65] Anderson ST, Kidd LJ, Benvenutti MA, Fletcher MT, Dixon RM. New candidate markers of phosphorus status in beef breeder cows. Animal Production Science. 2017;57(11):2291–2303. 10.1071/AN17363.

[66] Delmas P, Eastell R, Garnero P, Seibel M, Stepan J, Foundation CoSAotIO. The use of biochemical markers of bone turnover in osteoporosis. Osteoporosis International. 2000;11(6):S2–S17. 10.1007/s001980070002 11193237

[67] Sato R, Onda K, Kato H, Ochiai H, Kawai K, Iriki T, et al. An evaluation of the effect of age and the peri-parturient period on bone metabolism in dairy cows as measured by serum bone-specific alkaline phosphatase activity and urinary deoxypyridinoline concentration. The Veterinary Journal. 2013;197(2):358–362. 10.1016/j.tvjl.2013.01.013 23422881

[68] Kurosaki N, Yamato O, Sato J, Naito Y, Mori F, Imoto S, et al. Biomarkers for the Activation of Calcium Metabolism in Dairy Cows: Elevation of Tartrate-Resistant Acid Phosphatase Activity by Lowering Dietary Cation-Anion Difference is Associated with the Prevention of Milk Fever. Journal of Veterinary Medical Science. 2007;69(3):265–270. 10.1292/jvms.69.265 17409642

[69] Van Hoof V, Hoylaerts M, Geryl H, Van Mullem M, Lepoutre L, De Broe M. Age and sex distribution of alkaline phosphatase isoenzymes by agarose electrophoresis. Clinical chemistry. 1990;36(6):875–878. 2357825

[70] Delmas PD, Schlemmer A, Gineyts E, Riis B, Christiansen C. Urinary excretion of pyridinoline crosslinks correlates with bone turnover measured on iliac crest biopsy in patients with vertebral osteoporosis. Journal of Bone and Mineral Research. 1991;6(6):639–644. 10.1002/jbmr.5650060615 1887826

[71] Moreira VR, Zeringue LK, Williams CC, Leonardi C, McCormick ME. Influence of calcium and phosphorus feeding on markers of bone metabolism in transition cows. Journal of Dairy Science. 2009;92(10):5189–5198. 10.3168/jds.2009-2289 19762837

[72] Butler WR. Nutritional interactions with reproductive performance in dairy cattle. Animal Reproduction Science. 2000;60–61:449–457. 10.1016/s0378-4320(00)00076-2 10844215

[73] Bassett JD, Williams GR. Role of thyroid hormones in skeletal development and bone maintenance. Endocrine reviews. 2016;37(2):135–187. 10.1210/er.2015-1106 26862888 PMC4823381

[74] Silva JE. Thermogenic Mechanisms and Their Hormonal Regulation. Physiological Reviews. 2006;86(2):435–464. 10.1152/physrev.00009.2005 16601266

[75] van der Spek AH, Fliers E, Boelen A. The classic pathways of thyroid hormone metabolism. Molecular and Cellular Endocrinology. 2017. 10.1016/j.mce.2017.01.025 28109953

[76] Shao YY, Wang L, Ballock RT. Thyroid hormone and the growth plate. Reviews in Endocrine and Metabolic Disorders. 2007;7(4):265–271. 10.1359/jbmr.070806 17200892

[77] Glass AR, Mellitt R, Burman KD, Wartofsky L, Swerdloff RS. Serum triiodothyronine in undernourished rats: Dependence on dietary composition rather than total calorie or protein intake. Endocrinology. 1978;102(6):1925–1928. 10.1210/endo-102-6-1925 . 744060

[78] Sawaya AL, Lunn P. Evidence suggesting that the elevated plasma triiodothyronine concentration of rats fed on protein deficient diets is physiologically active. British journal of nutrition. 1985;53(01):175–181. 10.1079/bjn19850021 3904823

[79] Passos MCF, Ramos CF, Mouço T, Moura EG. Increase of T3 secreted through the milk in protein restricted lactating rats. Nutrition Research. 2001;21(6):917–924. 10.1016/S0271-5317(01)00294-9.

[80] Ramos CF, Teixeira CV, Passos MCF, Pazos-Moura CC, Lisboa PC, Curty FH, et al. Low-Protein Diet Changes Thyroid Function in Lactating Rats. Proceedings of the Society for Experimental Biology and Medicine. 2000;224(4):256–263. 10.1046/j.1525-1373.2000.22429.x 10964260

[81] Smallridge RC, Glass AR, Wartofsky L, Latham KR, Burman KD. Investigations into the etiology of elevated serum T3 levels in protein-malnourished rats. Metabolism. 1982;31(6):538–542. 10.1016/0026-0495(82)90091-9 6281617

[82] Isaksson OGPL, Nilsson A., and Isgaard J. Mechanism of the Stimulatory Effect of Growth Hormone on Longitudinal Bone Growth. Endocrine Reviews. 1987;8(4):426–438. 10.1210/edrv-8-4-426 . 3319530

[83] Naranjo WM, Yakar S, Sanchez-Gomez M, Perez AU, Setser J, LeRoith D. Protein Calorie Restriction Affects Nonhepatic IGF-I Production and the Lymphoid System: Studies Using the Liver-Specific IGF-I Gene-Deleted Mouse Model. Endocrinology. 2002;143(6):2233–2241. 10.1210/endo.143.6.8852 . 12021187

[84] Govoni KE, Lee SK, Chung Y-S, Behringer RR, Wergedal JE, Baylink DJ, et al. Disruption of insulin-like growth factor-I expression in type IIαI collagen-expressing cells reduces bone length and width in mice. Physiological Genomics. 2007;30(3):354–362. 10.1152/physiolgenomics.00022.2007 17519362 PMC2925693

[85] Sheng MHC, Zhou X-D, Bonewald LF, Baylink DJ, Lau KHW. Disruption of the insulin-like growth factor-1 gene in osteocytes impairs developmental bone growth in mice. Bone. 2013;52(1):133–144. 10.1016/j.bone.2012.09.027 23032105

