# Supplementary material for: Effect of a high crude protein content diet during energy restriction and re-alimentation on animal performance, skeletal growth and metabolism of bone tissue in two genotypes of cattle
Source: PLoS One. 2021 Feb 25;16(2):e0247718. doi: 10.1371/journal.pone.0247718 (PMC7906379; doi:10.1371/journal.pone.0247718)
Supplement: S1 Appendix — (DOCX) [file pone.0247718.s001.docx]

## S1 Appendix

The concentration of plasma glucose, nonesterified fatty acids (NEFA), calcium, inorganic phosphorus, urea (PUN) and total protein were measured using an Olympus AU400 auto-analyser (Beckman Coulter Diagnostic Systems Division, Melville, NY, USA). The concentration of insulin and IGF-1 in the plasma were determined by IRMA assays (DIAsource INS-IRMA Kit, DIAsource; Louvain-la-Neuve, Belgium; A15729, Immunotech, Beckman Coulter; Prague, Czech Republic). The inter- and intra-assay coefficients of variation were 9.7, 3.3% and 8.4 and 5.2%, for insulin and IGF-1 respectively. Total triiodothyronine (T3), Total thyroxine (T4) and leptin concentration in the plasma were analysed using RIA kits (IM1699 and IM1447, Immunotech, Beckman Coulter; Prague, Czech Republic; XL-85K, Millipore Corporation; St Charles, MO, USA). The inter- and intra assay coefficients of variation for these analyses were 5.3 and 6.4%, 3.7 and 7.2% and 6.0 and 4.2%, for T3, T4 and leptin respectively.

For all bone marker assays optical density of wells was determined using a plate reader spectrometer (Sunrise Absorbance Microplate Reader, Tecan; Pheonix, CA, USA) with XFluor software (Tecan; Pheonix, CA, USA). Bone-specific alkaline phosphatase (BALP) in plasma was determined by enzyme immunoassays (EIA) kit (MicroVue BAP 8012, Quidel; San Diego, CA, USA) and the inter- and intra-assay coefficient of variation were 3.6 and 4.2 for a quality control of 53.9 U/L. Osteocalcin (OCN) concentration in the plasma was measured using an EIA kit (MicroVue 8002 Osteocalcin, Quidel; San Diego, CA, USA) and the inter- and intra- assay coefficient of variation were 6.6% and 2.7% (respectively) for a quality control of 126.7 ng/mL. Pyridinoline Crosslinks (PYD) was determined using an EIA kit (MicroVue 8019 Serum PYD, Quidel; San Diego, CA, USA) and the inter and intra-assay coefficients of variation were 7.8% and 4.6% (respectively) for a quality control of 5.5 nmol/L. Total Deoxypyridinoline Crosslink (tDPD) in the plasma was determined using an EIA kit (MicroVue 8032 Total DPD, Quidel; San Diego, CA, USA) and the inter- and intra- assay coefficients of variation were 8.6% and 4.6% (respectively) for a quality control of 16.4 nmol/L. C-terminal Telopeptides of Type I Collagen (CTX-1) was determined using an EIA kit (Immunodiagnostic Systems Ltd; Boldon, UK) and the inter- and intra-assay coefficients of variation were 2.9% and 5.8% (respectively) for a quality control of 2.4 ng/mL.
